# Analytical Models for Multipath and Switch Leakage for the SWOT Interferometer

**DOI:** 10.3390/s22051931

**Published:** 2022-03-01

**Authors:** Razi Ahmed, Daniel Esteban-Fernández, Scott Hensley

**Affiliations:** Jet Propulsion Laboratory, California Institute of Technology, M/S 300-235, Pasadena, CA 91109, USA; daniel.esteban-fernandez@jpl.nasa.gov (D.E.-F.); shensley@jpl.nasa.gov (S.H.)

**Keywords:** radar, SAR interferometry, multipath phase, ocean topography

## Abstract

The Ka-Band Radar Interferometer (KaRIn) instrument on the Surface Water and Ocean Topography (SWOT) mission is a single-pass synthetic aperture radar (SAR) interferometer tasked with, among others, measuring ocean topography to within a few centimeters over kilometer scale resolutions. A SAR interferometer relies on very precise phase difference measurements between two spatially distant antennas to estimate topography. Multipath phase caused by unintended scattering off the spacecraft structure is a known error source for radar interferometers and takes up a significant portion of the KaRIn error budget. This paper outlines some analytical multipath models that were used for instrument design, performance analysis and mitigation of the multipath signal.

## 1. Introduction

The planned SWOT observatory [1,2,3,4], a collaboration between the US and French space agencies slated for launch in 2022, is designed to make high resolution, accurate two-dimensional measurements of ocean topography using SAR interferometry [5,6,7,8]. This unprecedented set of measurements would allow characterization of ocean mesoscale and sub-mesoscale dynamic processes [9,10,11] that are crucial to understanding and quantifying the exchange of energy between ocean layers and their impact on local and global carbon and energy budgets as well as nutrient transport [12,13]. The primary instrument on board the SWOT observatory enabling this unique set of measurements is KaRIn [14,15], a Ka-band (35 GHz) near-nadir radar interferometer capable of measuring sea-surface height with centimetric accuracy at kilometer scale resolutions [16], complemented with a nadir altimeter [17], GPS and DORIS [18] receivers and a three-frequency radiometer [19].

**Figure 1:**
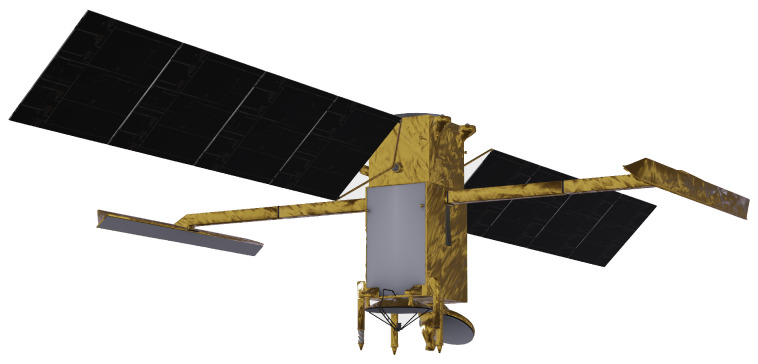
Structural model [29] of the SWOT Spacecraft with deployed KaRIn antennas and solar arrays and nadir altimeter/radiometer antenna pointing down.

The SWOT observatory, as seen in its deployed configuration in Figure 1, consists of a payload module containing the radar and subsystem electronics, the nadir module with nadir altimeter and radiometer antenna and solar arrays oriented perpendicular to the KaRIn 10 m interferometric baseline. The two rectangular Ka-band reflectarray antennas [20] (5×0.26 m) are mounted at the ends of two rigid masts, each approximately 4.3 m long. The two masts and reflectarrays are folded and stowed during launch and deploy once the spacecraft is in orbit [21]. Each reflectarray illuminates two swaths on either side of the nadir track by creating two beams using vertically displaced H- and V-polarized feeds located in the payload module, approximately 4.3 m from the reflectarrays. The centimeter scale sea surface height estimation requirement translates to very strict constraints on distortion of this structure (on the order of tens of microns). Consequently, the KaRIn mechanical structure is designed to be very rigid [20,22] and insulated from the environment using multi-layer insulation (MLI). MLI unfortunately is highly reflective at Ka-band frequencies and can change shape appreciably with temperature.

Single-pass interferometric SAR phase measurements, such as those from KaRIn, are often contaminated with systematic distortions such as multipath and switch leakage [23,24] that can affect the reconstructed height accuracy on the order of meters [25]. Multipath is caused by the transmit or receive signal bouncing off different parts of the spacecraft structure and coherently adding with the intended direct-path signal used for estimating topographic height, while switch leakage broadly refers to any signal leakage inside the radar electronics between the two interferometric channels. Provided these effects remain stable, they are typically calibrated out using a phase screen estimated during instrument calibration [24,26]. However, any variation in the multipath or leakage phase in addition to the static phase screen cannot be calibrated and thus contaminates height estimates directly. To ensure accurate height estimates, this variability needs to be mitigated during instrument design, requiring detailed analysis of electromagnetic scattering off the spacecraft structure and its impact on the interferometric measurement. Here, we present first order analytical models, similar to those discussed in the context of airborne single-pass SAR interferometers [27,28], to help assess the impact of multipath or leakage on KaRIn. The basic interferometric measurement [8], γ, is a product of the two complex voltages received at the two antennas, $s_{1}$ and $s_{2}$, given by

(1) $$\begin{array}{c} \gamma =\frac{\langle s_{1}s_{2}^{*}\rangle }{\sqrt{\langle {\left|s_{1}\right|}^{2}\rangle \langle {\left|s_{2}\right|}^{2}\rangle }}. \end{array}$$

The interferometric phase is defined as $\phi =\arg\left\{\gamma \right\}$, which is ideally $\phi =k\left(\rho_{2}-\rho_{1}\right)$, where $\rho_{1}$ and $\rho_{2}$ are the range to the imaged target from antennas 1 and 2, respectively. However, due to multipath and leakage, the measured phase is really

(2) $$\begin{array}{c} \phi_{m}=\arg\left\{\gamma_{m}\right\}=\phi +\phi_{s} \end{array}$$

where $\phi_{s}$ is referred to as the phase screen that includes the effects of multipath and leakage. Phase errors such as this can be converted into height errors using

(3) $$\begin{array}{c} \Delta h=\frac{\partial h}{\partial \phi }\phi_{s}=\frac{\phi_{s}}{k_{z}} \end{array}$$

with the $k_{z}$, the vertical wavenumber, given approximately by [8]

(4) $$\begin{array}{c} k_{z}=\frac{2\pi B_{⊥}}{\lambda \rho \sin\theta } \end{array}$$

where $B_{⊥}$ is the interferometric baseline (perpendicular to the look direction), λ is the radar wavelength (8.4 mm for SWOT), ρ the slant range and θ the look angle (varying between 0.5° and 5°). For SWOT geometry, $k_{z}$ varies between 0.1016 rad/m in the near swath to 1.3175 rad/m in the far swath. The very strict height measurement requirements of SWOT meant multipath height must not vary more than a few mm, resulting in $\Delta \phi_{s}$ to be limited to below 6.5 mdeg. This very tight requirement led to an in-depth analysis of the multipath issue including electromagnetic simulations and thermoelastic modeling of the spacecraft structure, among others. While specific details of that study are outside the scope of this article and of limited use to the general community, the authors hope that analytical models of $\phi_{s}$ presented in this article for various scenarios applicable to SWOT are generic enough to be of broader use.

## 2. Signal Leakage

**Figure 2:**
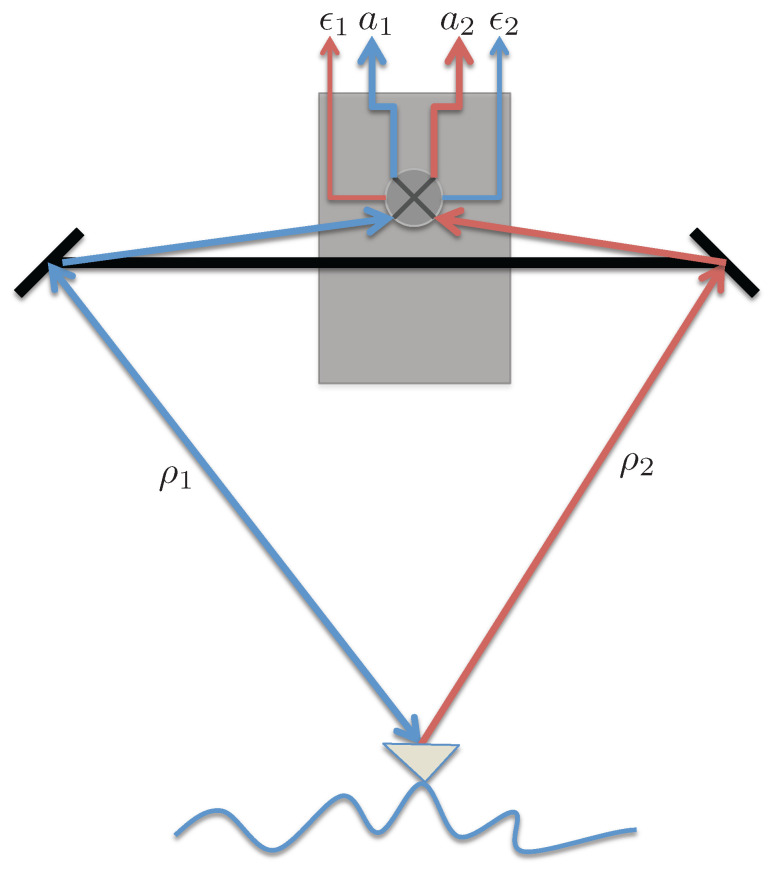
Illustration of received signal paths for a scenario with signal leakage through the RF electronics.

To begin with, a simple model for signal leakage is developed by assuming that the signal received at either antenna leaks into the other channel through the RF electronics as illustrated by Figure 2.

The signal received at the left antenna (for this discussion, let us assume that is the transmit antenna in a bistatic configuration) can be written as [8]

(5) $$\begin{array}{c} s_{1}=a_{1}e^{-j2k\rho_{1}}+\epsilon_{1}e^{-jk\left(\rho_{1}+\rho_{2}\right)} \end{array}$$

where $a_{1}$ is the amplitude of the desired signal which travels a total distance of $2\rho_{1}$ (to and from the imaged object), while the undesired signal leaks through the RF electronics with an amplitude of $\epsilon_{1}$ and arrives after traveling a total distance of $\rho_{1}+\rho_{2}$ with k=2π/λ is typically referred to as the wavenumber. Similarly, the signal received at the second (right) channel can be written as

(6) $$\begin{array}{c} s_{2}=a_{1}e^{-jk\left(\rho_{1}+\rho_{2}\right)}+\epsilon_{2}e^{-j2k\rho_{1}}. \end{array}$$

The interferometric measurement is given by

(7) $$\begin{array}{cc} \gamma & =s_{1}s_{2}^{*}\\ \; & =\left(a_{1}e^{-j2k\rho_{1}}+\epsilon_{1}e^{-jk\left(\rho_{1}+\rho_{2}\right)}\right)\left(a_{1}e^{jk\left(\rho_{1}+\rho_{2}\right)}+\epsilon_{2}e^{j2k\rho_{1}}\right) \end{array}$$

which can be written as a product of the desired interferometric signal and the leakage as

(8) $$\begin{array}{cc} \gamma & =a_{1}^{2}e^{-jk\left(\rho_{1}-\rho_{2}\right)}\left\{1+\frac{\epsilon_{1}+\epsilon_{2}}{a_{1}}e^{jk\left(\rho_{1}-\rho_{2}\right)}+\frac{\epsilon_{1}\epsilon_{2}}{a_{1}^{2}}e^{-2jk\left(\rho_{1}-\rho_{2}\right)}\right\}. \end{array}$$

Assuming $\frac{\epsilon_{1}\epsilon_{2}}{a_{1}^{2}}\ll \frac{\epsilon_{1}+\epsilon_{2}}{a_{1}}$, the above expression simplifies to

(9) $$\begin{array}{cc} \gamma & \approx a_{1}^{2}e^{-jk\left(\rho_{1}-\rho_{2}\right)}\left\{1+\frac{\epsilon_{1}+\epsilon_{2}}{a_{1}}e^{jk\left(\rho_{1}-\rho_{2}\right)}\right\}. \end{array}$$

Since the interferometric phase, $\phi =\arg\left(\gamma \right)$, can then be written as a sum of desired and undesired (leakage) phase as $\phi =\phi_{d}+\phi_{l}$, the leakage phase can be derived trivially (following for instance Appendix A) as

(10) $$\begin{array}{c} \phi_{l}=\tan^{-1}\left[\frac{\frac{\epsilon_{1}+\epsilon_{2}}{a_{1}}\sin\left(kb\sin\left(\theta -\alpha \right)\right)}{1+\frac{\epsilon_{1}+\epsilon_{2}}{a_{1}}\cos\left(kb\sin\left(\theta -\alpha \right)\right)}\right] \end{array}$$

where θ is the look angle, α is the baseline roll angle and *b* is the baseline length (10 m for SWOT). This simplified model for switch leakage can be used to either determine requirements on channel isolation or to help identify contribution of leakage in the measured interferometric phase.

## 3. Multipath

Radar signals can bounce off different parts of the spacecraft and add up coherently to corrupt the interferometric phase measurement. One can think of a multitude of multipath scenarios with structures as complicated as SWOT. While it is not possible to give a full accounting of these effects using simple analytical expressions, a few likely scenarios can in any case be looked at in isolation to develop an intuition of the effects of multipath scattering on the measured interferometric phase.

### 3.1. Multipath between the Two Interferometric Antennas

**Figure 3:**
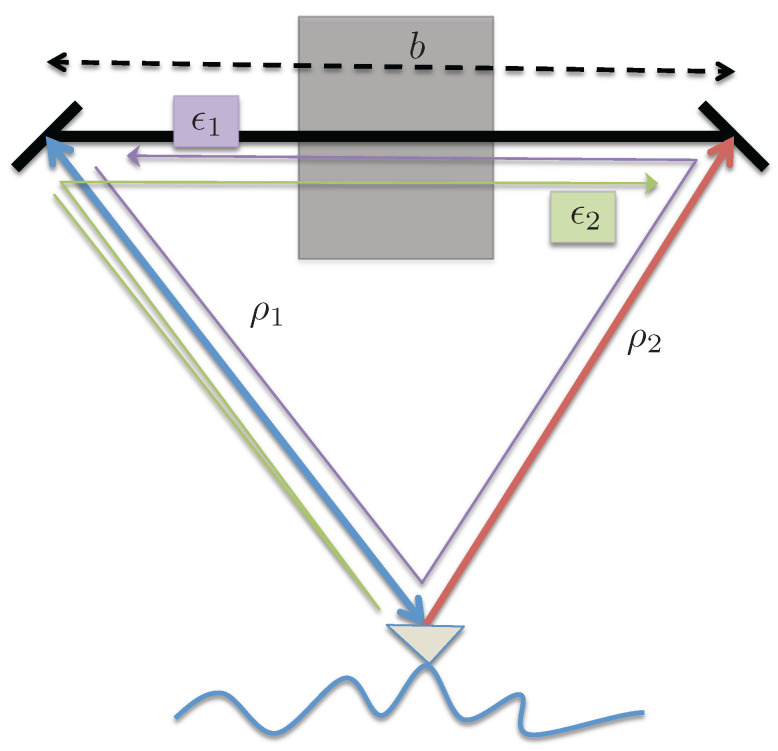
Illustration of signal paths for a multipath scenario with scattered signal bouncing off the other antenna. The desired signal paths are shown with the thicker lines; blue line for the signal path from the transmitting antenna to the surface and back, and red line for the second, receive-only antenna. The thinner lines show the same paths but include scattering from one antenna to the other as the undesired, or multipath, signal.

Consider that the received signal at the respective antenna bounces off and is received by the other aperture after traveling an additional path length (equal to the interferometric baseline). The signal paths for this scenario are illustrated in Figure 3.

The signals received at the two antennas can be written as

(11) $$\begin{array}{cc} s_{1} & =a_{1}e^{-j2k\rho_{1}}+\epsilon_{1}e^{-jk\left(\rho_{1}+\rho_{2}+b\right)}\\ s_{2} & =a_{1}e^{-jk\left(\rho_{1}+\rho_{2}\right)}+\epsilon_{2}e^{-jk\left(2\rho_{1}+b\right)} \end{array}$$

where *b* is the interferometric baseline length. The interferometric measurement $\gamma =s_{1}s_{2}^{*}$ can be written as

(12) $$\begin{array}{cc} \gamma & =a_{1}^{2}e^{-jk\left(\rho_{1}-\rho_{2}\right)}\left\{1+\frac{\epsilon_{1}e^{jkb}+\epsilon_{2}e^{-jkb}}{a_{1}}e^{jk\left(\rho_{1}-\rho_{2}\right)}+\frac{\epsilon_{1}\epsilon_{2}}{a_{1}^{2}}e^{-2jk\left(\rho_{1}-\rho_{2}\right)}\right\}. \end{array}$$

Assuming $\epsilon_{1}\epsilon_{2}/a_{1}^{2}\ll \left(\epsilon_{1}+\epsilon_{2}\right)/a_{1}$, the above expression simplifies to

(13) $$\begin{array}{cc} \gamma & =a_{1}^{2}e^{-jk\left(\rho_{1}-\rho_{2}\right)}\left\{1+\left(\frac{2\epsilon_{1}}{a_{1}}\cos\left(kb\right)+\frac{\epsilon_{2}-\epsilon_{1}}{a_{1}}e^{jkb}\right)e^{jk\left(\rho_{1}-\rho_{2}\right)}\right\}. \end{array}$$

The multipath phase from the other antenna can then be written as

(14) $$\begin{array}{c} \phi_{mp}=\tan^{-1}\left\{\frac{2\epsilon_{1}\sin\beta_{1}+\left(\epsilon_{2}-\epsilon_{1}\right)\sin\beta_{2}}{a_{1}+2\epsilon_{1}\cos\left(kb\right)\cos\beta_{1}+\left(\epsilon_{2}-\epsilon_{1}\right)\cos\beta_{2}}\right\}. \end{array}$$

where $\beta_{1}=kb\sin\left(\theta -\alpha \right)$, and $\beta_{2}=\beta +kb$.

**Figure 4:**
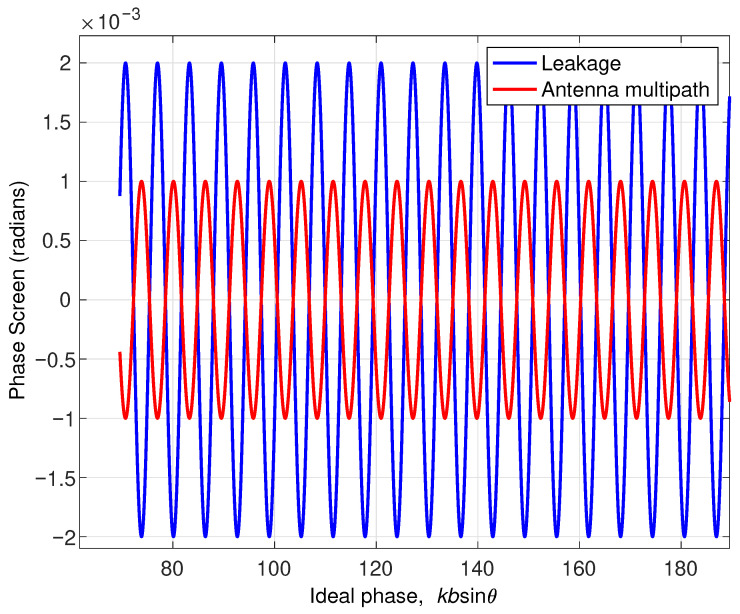
An example of phase distortion due to RF leakage and antenna multipath using a 60 dB signal to leakage ratio assuming $\epsilon_{1}=\epsilon_{2}$.

**Figure 5:**
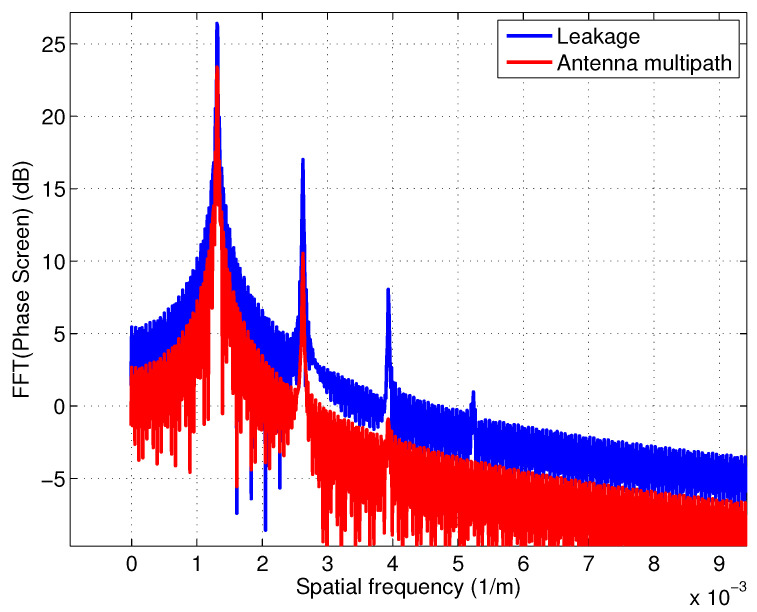
Spectral components of phase distortion due to RF leakage and antenna multipath using a 60 dB signal to leakage ratio assuming $\epsilon_{1}=\epsilon_{2}$.

Figure 4 shows the effect of RF leakage and antenna multipath as a function of *ideal phase* (kbsinθ) on the interferometric phase for the SWOT geometry and Figure 5 shows the spectral components of interferometric phase for both cases. The two cases appear to have similar shape (albeit out of phase) and contain the same spectral signatures. For SWOT, this scenario is unlikely as the two antennas are obscured by the payload module.

### 3.2. Multipath from One Specular Point on the Transmitter Side

**Figure 6:**
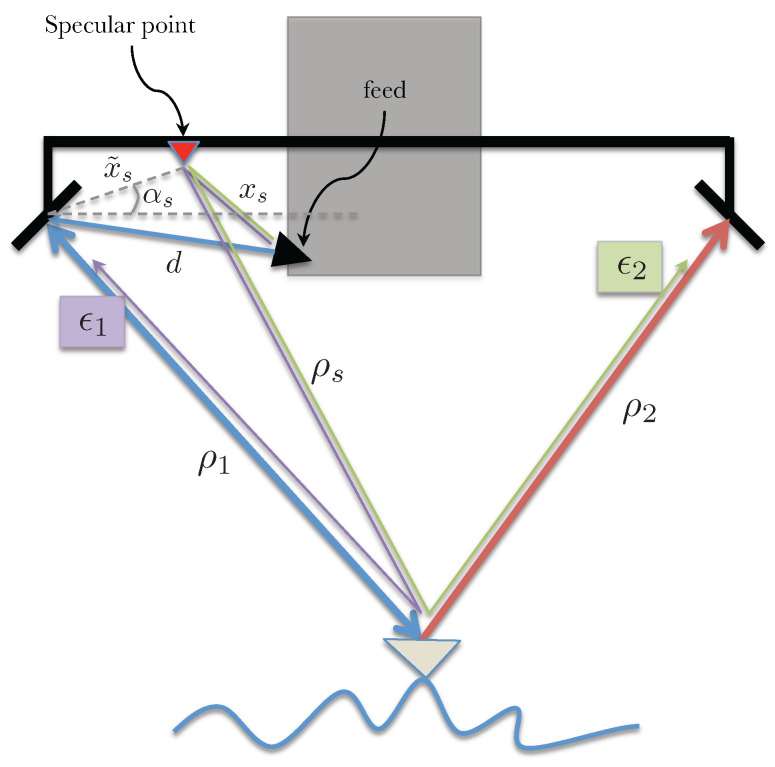
Illustration of received signal paths for a multipath scenario with one specular point acting as a source.

In this scenario, consider one particular point between the transmit reflectarray and the feed, e.g., the transmit signal from the feed bounces off some part of the mast, as illustrated in Figure 6, to the imaged object and the return energy adds up coherently with the desired signal at the reflectarray. This multipath signal does not have the gain of the main reflectarray and is expected to be very small; however, since SWOT requirements on phase stability are very strict, it is important to assess the impact of this plausible multipath scenario.

The received fields at the two reflectarrays can be written as

(15) $$\begin{array}{cc} s_{1} & =a_{1}e^{-jk\left(d+2\rho_{1}\right)}+2\epsilon_{1}e^{-jk\left(x_{s}+\rho_{s}+\rho_{1}+\phi_{s}/k\right)}\\ s_{2} & =a_{1}e^{-jk\left(d+\rho_{1}+\rho_{2}\right)}+\epsilon_{2}e^{-jk\left(x_{s}+\rho_{s}+\rho_{2}+\phi_{s}/k\right)} \end{array}$$

where *d* is the distance between the transmit feed and the transmit side reflectarray, $x_{s}$ is the distance between the specular point and the transmit reflectarray and $\phi_{s}$ is a phase term introduced by the specular point (this additional phase term is expected to be a function of the look angle). Note the factor of 2 next to $\epsilon_{1}$ is due to the two equivalent signal paths, one where the specular point acts as the transmitter and the reflectarray as the receiver and the second where the reflectarray transmits and the specular point acts as the receiver. The range of the imaged object from the specular point and the reflectarrays are $\rho_{s}$ and $\rho_{i}$ (for i=1,2, i.e., the left and right reflectarrays), respectively. Assuming $\epsilon_{1}\epsilon_{2}/a_{1}^{2}\ll \left(\epsilon_{1}+\epsilon_{2}\right)/a_{1}$, the interferometric measurement $\gamma =s_{1}s_{2}^{*}$ can be written as

(16) $$\begin{array}{c} \gamma =a_{1}^{2}e^{-jk\left(\rho_{1}-\rho_{2}\right)}\left\{1+\frac{2\epsilon_{1}}{a_{1}}e^{-jk\left(d-x_{s}+\rho_{1}-\rho_{s}-\phi_{s}/k\right)}+\frac{\epsilon_{2}}{a_{1}}e^{jk\left(d-x_{s}+\rho_{1}-\rho_{s}-\phi_{s}/k\right)}\right\} \end{array}$$

Since $\rho_{1}-\rho_{s}={\tilde{x}}_{s}\sin\left(\theta -\alpha_{s}\right)$, where $\alpha_{s}$ is the angle subtended by ${\tilde{x}}_{s}$ measured from the horizontal. The multipath phase screen can then be written as

(17) $$\begin{array}{c} \phi_{mp}=\tan^{-1}\left\{\frac{\left(2\epsilon_{1}-\epsilon_{2}\right)\sin\beta }{a_{1}+\left(2\epsilon_{1}+\epsilon_{2}\right)\cos\beta }\right\}. \end{array}$$

where $\beta =k\left(d-x_{s}-\phi_{s}/k+{\tilde{x}}_{s}\sin\left(\theta -\alpha_{s}\right)\right)$. Multipath phase in this case varies across swath as a function of the look angle, mostly sinusoidally, with its frequency depending on the distance of the scattering point from the antenna (longer distances leading to higher spatial frequencies). The amplitude of the phase screen here is dependent on the difference between the received multipath voltage at the two antennas.

#### Multipath Form More than One Specular Point on the Transmit Side

Instead of having only one specular point, it is possible that the signal from the transmit feed is incident on more than one specular point simultaneously. Each specular point, in such a case, would add to the phase screen coherently but at a different spatial frequency. For *N* specular points, the received fields can be written as

(18) $$\begin{array}{cc} s_{1} & =a_{1}e^{-jk\left(d+2\rho_{1}\right)}+2\sum_{i}^{N}\epsilon_{1_{i}}e^{-jk\left(x_{s_{i}}+\rho_{s_{i}}+\rho_{1}+\phi_{s_{i}}/k\right)}\\ s_{2} & =a_{1}e^{-jk\left(d+\rho_{1}+\rho_{2}\right)}+\sum_{i}^{N}\epsilon_{2_{i}}e^{-jk\left(x_{s_{i}}+\rho_{s_{i}}+\rho_{2}+\phi_{s_{i}}/k\right)} \end{array}$$

where $x_{s_{i}}$ is the distance between the feed and the *i*th specular point (shown as the distance $x_{s}$ in Figure 6 for the case with only one specular point) and similarly $\rho_{s_{i}}$ is the distance from the *i*th specular point to the imaged object while $\phi_{s_{i}}$ is the look angle dependent phase introduced by the *i*th specular point. With the usual assumptions, the phase screen in this case can be written as

(19) $$\begin{array}{c} \phi_{mp}=\tan^{-1}\left\{\frac{\sum_{i}^{N}\left(2\epsilon_{1_{i}}-\epsilon_{2_{i}}\right)\sin\beta_{i}}{a_{1}+\sum_{i}^{N}\left(2\epsilon_{1_{i}}+\epsilon_{2_{i}}\right)\cos\beta_{i}}\right\} \end{array}$$

where $\beta_{i}=k\left(d-x_{s_{i}}-\phi_{s_{i}}/k+{\tilde{x}}_{s_{i}}\sin\left(\theta -\alpha_{s_{i}}\right)\right)$ and $\alpha_{s_{i}}$ is the roll angle of ${\tilde{x}}_{s_{i}}$ relative to the horizontal.

**Figure 7:**
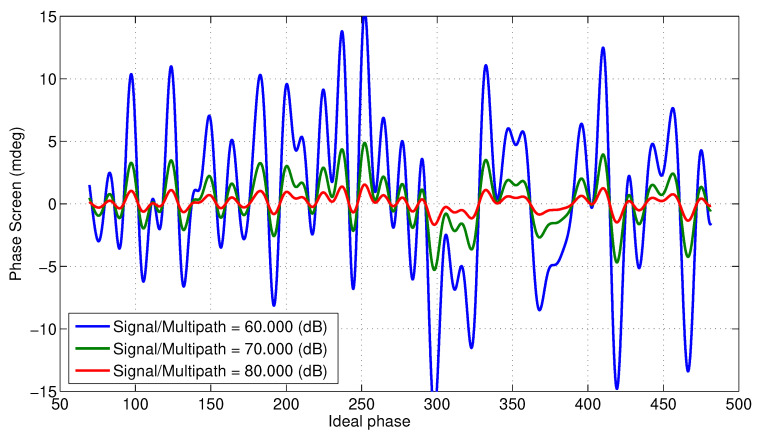
Expected phase screen for various signal to multipath ratios with specular points spread evenly along the mast, assuming $\epsilon_{2}=0$.

**Figure 8:**
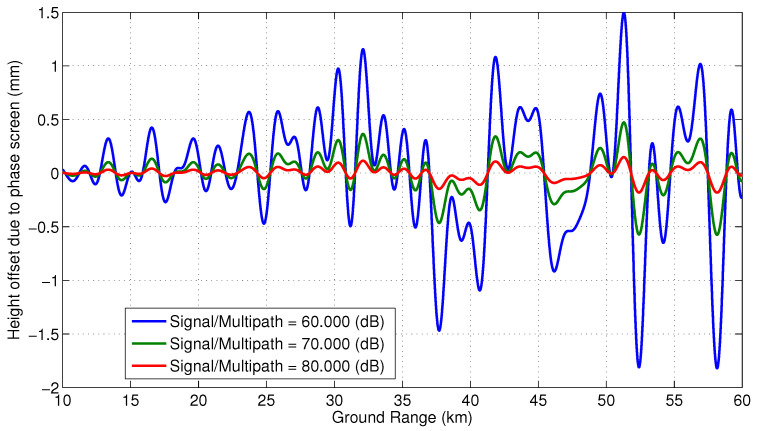
Expected height offset due to multipath from specular points spread evenly along the mast, assuming $\epsilon_{2}=0$.

Figure 7 shows expected phase screens with specular points spread evenly along the 4.3 m SWOT mast. This plot assumes signal to multipath ratios $\left(20\log\left(a_{1}/e_{1}\right)\right)$ of 60, 70 and 80 dB, with $\epsilon_{2}=0$ to simulate the worst case scenario. Figure 8 shows the effective height offsets across the swath due to multipath from specular points spread evenly along the mast.

**Figure 9:**
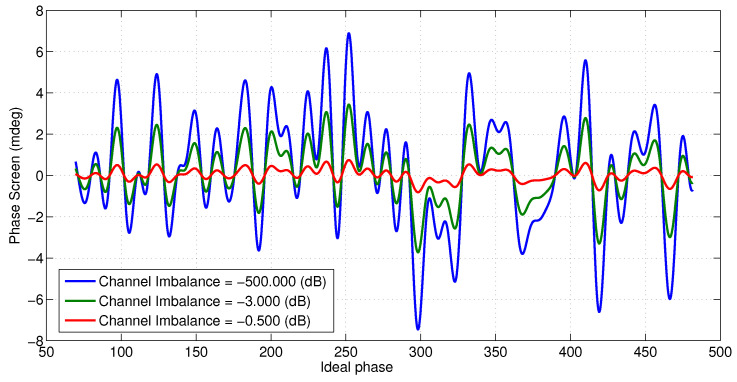
Expected phase screen for 67 dB signal to multipath ratio and varying channel imbalance with specular points spread evenly along the mast.

**Figure 10:**
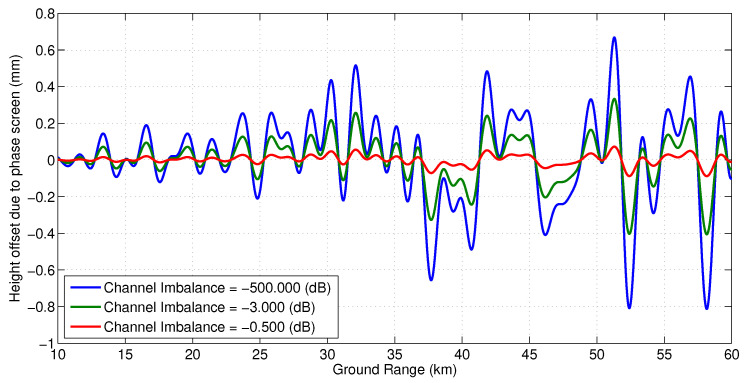
Expected height offset due to multipath from specular points spread evenly along the mast with 67 dB signal to multipath ratio.

**Figure 11:**
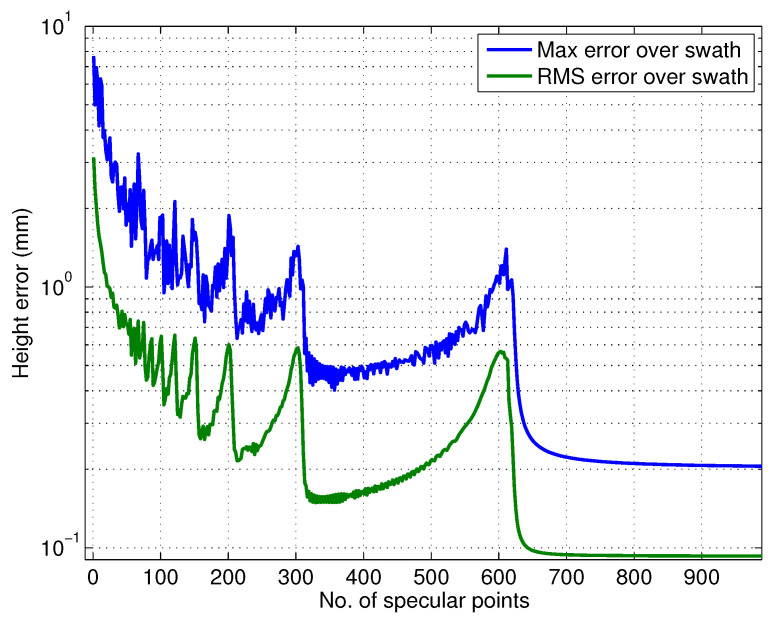
Expected height offset due to multipath from different number of specular points spread evenly along the mast with 57 dB signal to multipath ratio.

Figure 9 and Figure 10 show the effect of channel imbalance on the phase screen amplitude and height bias, respectively. Here, channel imbalance refers to the difference between the amplitude of the multipath signals received at the two channels, i.e., $\delta =10\log(\epsilon_{2}/\epsilon_{1})$. Figure 11 shows two statistics, the maximum height error in the swath and the total RMS height error across the swath as a function of varying number of specular points along the 4.3 m mast (spaced evenly) for an amplitude to multipath ratio of 57 dB.

Because of the complex cross-track shape, dependence on unknowable quantities such as channel imbalance and sensitivity to specular scatterer location, this type of multipath was determined to be the largest expected multipath error and also most likely to vary significantly with temperature. Structures were designed and installed on the SWOT spacecraft to reduce this type of multipath scattering.

### 3.3. Multipath between the Feed and the Reflectarray

**Figure 12:**
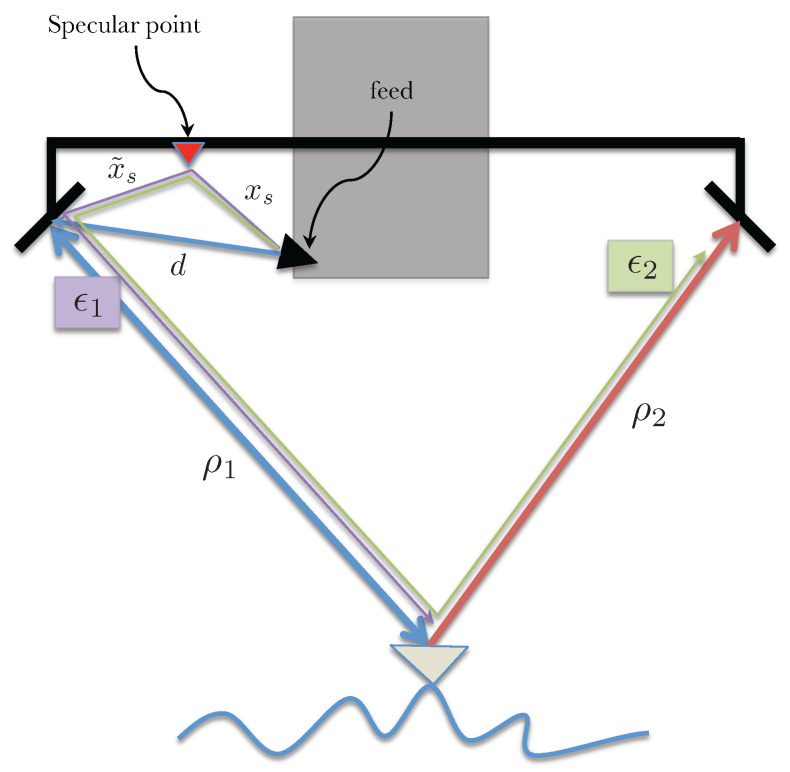
Illustration of received signal paths for a multipath scenario with a specular point between the feed and the reflectarray.

Now, consider a scenario where the multipath occurs between the feed and the reflectarray of the transmit antenna. Figure 12 highlights the geometry of one specular point between the feed and the reflectarray and the associated paths for the signals.

In this scenario, the signals received at the two antennas can be written as

(20) $$\begin{array}{cc} s_{1} & =a_{1}e^{-jk\left(d+2\rho_{1}\right)}+2\epsilon_{1}e^{-jk\left(x_{s}+{\tilde{x}}_{s}+2\rho_{1}+\phi_{s}/k\right)}\\ s_{2} & =a_{1}e^{-jk\left(d+\rho_{1}+\rho_{2}\right)}+\epsilon_{2}e^{-jk\left(x_{s}+{\tilde{x}}_{s}+\rho_{1}+\rho_{2}+\phi_{s}/k\right)} \end{array}$$

where ${\tilde{x}}_{s}$ is the direct path length between the reflectarray (the imaging point) and the feed, while *d* and $x_{s}$ are the path length between the feed and the specular point and between the specular point and the reflectarray, respectively. Assuming $\epsilon_{1}\epsilon_{2}/a_{1}^{2}\ll \left(\epsilon_{1}+\epsilon_{2}\right)/a_{1}$, the interferometric measurement $\gamma =s_{1}s_{2}^{*}$ can be written as

(21) $$\begin{array}{cc} \gamma & =a_{1}^{2}e^{-jk\left(\rho_{1}-\rho_{2}\right)}\left\{1+\frac{2\epsilon_{1}}{a_{1}}e^{-jk\left(x_{s}+{\tilde{x}}_{s}-d+\phi_{s}/k\right)}+\frac{\epsilon_{2}}{a_{1}}e^{jk\left(x_{s}+{\tilde{x}}_{s}+2\rho_{1}+\phi_{s}/k\right)}\right\}. \end{array}$$

The multipath phase, following the usual algebra, can then be written as

(22) $$\begin{array}{c} \phi_{mp}=\tan^{-1}\left\{\frac{\left(2\epsilon_{1}-\epsilon_{2}\right)\sin\beta }{a_{1}+\left(2\epsilon_{1}+\epsilon_{2}\right)\cos\beta )}\right\}. \end{array}$$

where $\beta =k\left(x_{s}+{\tilde{x}}_{s}+\phi_{s}/k-d\right)$. Multipath phase in this case will be constant across swath, while the amplitude will depend on the difference between the received multipath voltages and location of the specular point. This particular type of multipath scattering is preferable for SWOT since the cross-track variation is negligible and therefore more amenable to calibration than the scenario outlined in Section 3.2.

#### Multipath from Multiple Specular Points between the Feed and the Reflectarray

We can imagine having multiple specular points between the feed and the reflectarray as sources for multipath. For *N* specular points, the received fields can be written as

(23) $$\begin{array}{cc} s_{1} & =a_{1}e^{-jk\left(d+2\rho_{1}\right)}+2\sum_{i}^{N}\epsilon_{1_{i}}e^{-jk\left(x_{s_{i}}+{\tilde{x}}_{s_{i}}+2\rho_{1}+\phi_{s_{i}}/k\right)}\\ s_{2} & =a_{1}e^{-jk\left(d+\rho_{1}+\rho_{2}\right)}+\sum_{i}^{N}\epsilon_{2_{i}}e^{-jk\left(x_{s_{i}}+{\tilde{x}}_{s_{i}}+\rho_{1}+\rho_{2}+\phi_{s_{i}}/k\right)} \end{array}$$

where $x_{s_{i}}$ is the distance between the feed and the *i*th specular point, while ${\tilde{x}}_{s_{i}}$ is the distance between the *i*th specular point and the reflectarray. With the usual assumptions, the phase screen can be written as

(24) $$\begin{array}{c} \phi_{mp}=\tan^{-1}\left\{\frac{\sum_{i}^{N}\left(2\epsilon_{1_{i}}-\epsilon_{2_{i}}\right)\sin\beta_{i}}{a_{1}+\sum_{i}^{N}\left(2\epsilon_{1_{i}}+\epsilon_{2_{i}}\right)\cos\beta_{i}}\right\}. \end{array}$$

where $\beta_{i}=k\left(x_{s_{i}}+{\tilde{x}}_{s_{i}}+\phi_{s_{i}}/k-d\right)$. Without any particular cross-track shape in the multipath phase, it is hard to determine which particular spacecraft structure contributes more to multipath. Mechanically rigid structures are put in place on the SWOT spacecraft to reduce variation in this type of multipath, with the aim of calibrating the gross multipath phase using a phase screen.

### 3.4. Simultaneous Multipath Scenarios

**Figure 13:**
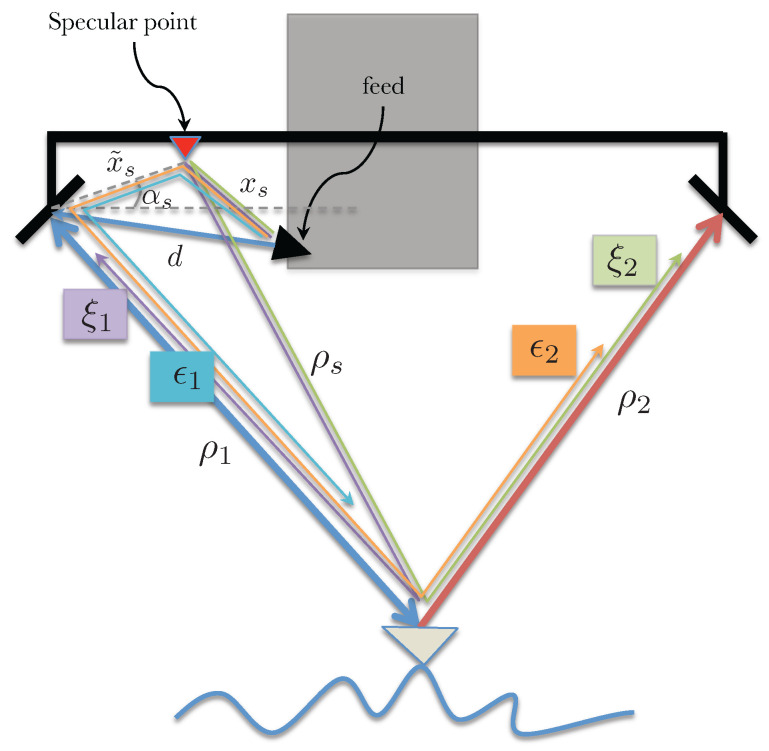
Illustration of received signal paths for two simultaneous multipath signals.

The previous sections have described the effects of multipath on the interferometric phase measurement as two separate scenarios, however, it is quite likely that these two scenarios occur simultaneously, and the multipath signals are of comparable magnitude. Figure 13 illustrates the geometry of the more general scenario with both types of multipath from the same specular point. In this case, as will be shown shorty, the effect on phase is not a straightforward superposition of the two cases.

The received signals in this case can be written as

(25) $$\begin{array}{cc} s_{1} & =a_{1}e^{-jk\left(d+2\rho_{1}\right)}+2\epsilon_{1}e^{-jk\left(x_{s}+{\tilde{x}}_{s}+2\rho_{1}+{\tilde{\phi }}_{s}/k\right)}+2\xi_{1}e^{-jk\left(x_{s}+\rho_{s}+\rho_{1}+\phi_{s}/k\right)}\\ s_{2} & =a_{1}e^{-jk\left(d+\rho_{1}+\rho_{2}\right)}+\epsilon_{2}e^{-jk\left(x_{s}+{\tilde{x}}_{s}+\rho_{1}+\rho_{2}+{\tilde{\phi }}_{s}/k\right)}+\xi_{2}e^{-jk\left(x_{s}+\rho_{s}+\rho_{2}+\phi_{s}/k\right)}. \end{array}$$

The interferometric measurement $\gamma =s_{1}s_{2}^{*}$ will have nine terms; however, assuming error product terms (i.e., $\epsilon_{1}\epsilon_{2}$, $\epsilon_{1}\xi_{1}$, $\epsilon_{2}\xi_{1}$, etc.) can be ignored, γ can be approximated as

(26) $$\begin{array}{cc} \gamma & =a_{1}^{2}e^{-jk\left(\rho_{1}-\rho_{2}\right)}+a_{1}\epsilon_{2}e^{-jk\left(d+2\rho_{1}-x_{s}-{\tilde{x}}_{s}-\rho_{1}-\rho_{2}-{\tilde{\phi }}_{s}/k\right)}\\ \; & \;\;+a_{1}\xi_{2}e^{-jk\left(d+2\rho_{1}-x_{s}-\rho_{s}-\rho_{2}-\phi_{s}/k\right)}\\ \; & \;\;+2a_{1}\epsilon_{1}e^{-jk\left(x_{s}+{\tilde{x}}_{s}+2\rho_{1}+{\tilde{\phi }}_{s}/k-d-\rho_{1}-\rho_{2}\right)}\\ \; & \;\;+2a_{1}\xi_{1}e^{-jk\left(x_{s}+\rho_{s}+\rho_{1}+\rho_{2}+\phi_{s}/k-d-\rho_{1}-\rho_{2}\right)}. \end{array}$$

Since $\rho_{1}-\rho_{s}={\tilde{x}}_{s}\sin\left(\theta -\alpha_{s}\right)$, where $\alpha_{s}$ is the angle subtended by ${\tilde{x}}_{s}$ measured from the horizontal, the multipath phase screen can then be written as

(27) $$\begin{array}{c} \phi_{mp}=\tan^{-1}\left\{\frac{\left(2\epsilon_{1}-\epsilon_{2}\right)\sin\psi +\left(2\xi_{1}-\xi_{2}\right)\sin\chi }{a_{1}+\left(2\epsilon_{1}+\epsilon_{2}\right)\cos\psi +\left(2\xi_{1}+\xi_{2}\right)\cos\chi }\right\} \end{array}$$

where $\psi =k\left(x_{s}+{\tilde{x}}_{s}+{\tilde{\phi }}_{s}/k-d\right)$ and $\chi =k\left(d-x_{s}+{\tilde{x}}_{s}\sin\left(\theta -\alpha_{s}\right)-\phi_{s}/k\right)$. As evident, the individual multipath scenarios appear as sums in the numerator and denominator of this equation, but the formulation is not a strict superposition of the two individual cases.

#### Simultaneous Multipath Scenarios from More than One Specular Point

Now, assume the most generic scenario considered so far, where more than one specular point act as both reflectarrays to the imaged object and between the feed and the reflectarray. The received fields for this case can be written as

(28) $$\begin{array}{cc} s_{1} & =a_{1}e^{-jk\left(d+2\rho_{1}\right)}+2\sum_{i}^{N}\epsilon_{1_{i}}e^{-jk\left(x_{s_{i}}+{\tilde{x}}_{s_{i}}+2\rho_{1}+{\tilde{\phi }}_{s_{i}}/k\right)}+2\sum_{i}^{N}\xi_{1_{i}}e^{-jk\left(x_{s_{i}}+\rho_{s_{i}}+\rho_{1}+\phi_{s_{i}}/k\right)}\\ s_{2} & =a_{1}e^{-jk\left(d+\rho_{1}+\rho_{2}\right)}+\sum_{i}^{N}\epsilon_{2_{i}}e^{-jk\left(x_{s_{i}}+{\tilde{x}}_{s_{i}}+\rho_{1}+\rho_{2}+{\tilde{\phi }}_{s_{i}}/k\right)}+\sum_{i}^{N}\xi_{2_{i}}e^{-jk\left(x_{s_{i}}+\rho_{s_{i}}+\rho_{2}+\phi_{s_{i}}/k\right)}. \end{array}$$

with the usual assumptions, the phase screen can be written as

(29) $$\begin{array}{cc} \phi_{mp} & =\tan^{-1}\left\{\frac{\sum_{i}^{N}\left(2\epsilon_{1_{i}}-\epsilon_{2_{i}}\right)\sin\psi_{i}+\sum_{i}^{N}\left(2\xi_{1_{i}}-\xi_{2_{i}}\right)\sin\chi_{i}}{a_{1}+\sum_{i}^{N}\left(2\epsilon_{1_{i}}+\epsilon_{2_{i}}\right)\cos\psi_{i}+\sum_{i}^{N}\left(2\xi_{1_{i}}+\xi_{2_{i}}\right)\cos\chi_{i}}\right\} \end{array}$$

where $\psi_{i}=k\left(x_{s_{i}}+{\tilde{x}}_{s_{i}}+{\tilde{\phi }}_{s_{i}}/k-d\right)$ and $\chi_{i}=k\left(d-x_{s_{i}}+{\tilde{x}}_{s_{i}}\sin\left(\theta -\alpha_{m_{i}}\right)-\phi_{s_{i}}/k\right)$. At this point, simple analytical models become increasingly cumbersome and less useful for developing an intuitive understanding of the multipath issue. There is, however, some limited value in using these equations as forward models to bound the expected error.

## 4. Verifying Multipath Models

**Figure 14:**
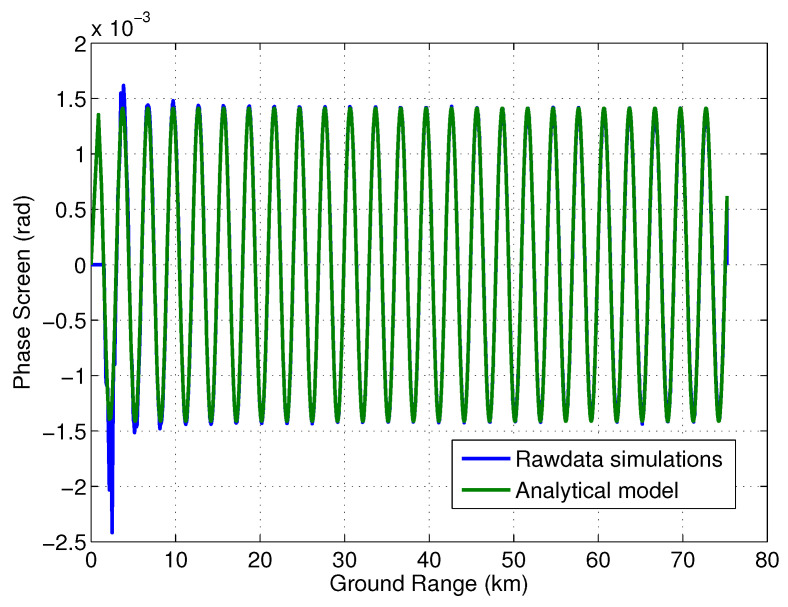
Simulated multipath plotted along with the phase screen model given in (30).

**Figure 15:**
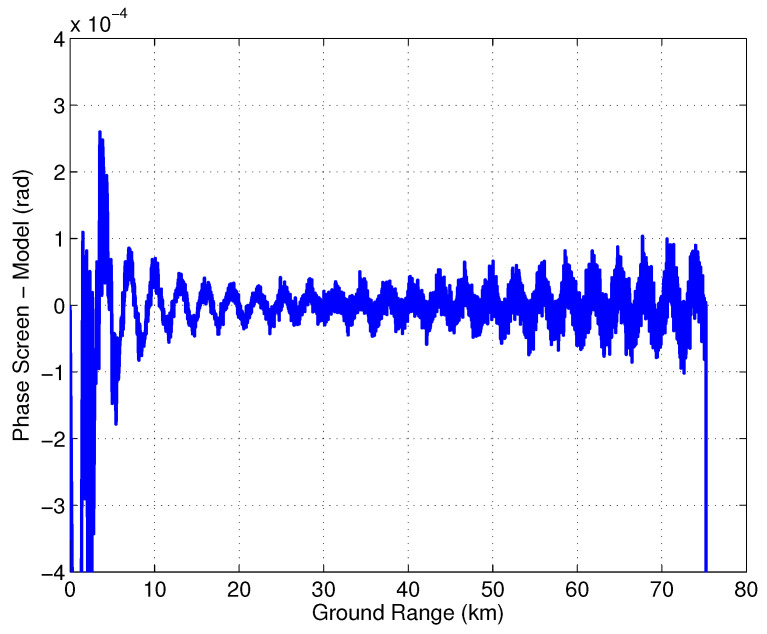
Difference between the simulated phase screen and the analytic model.

It is hard to fully verify multipath models; however, some select cases can be looked at as a verification of the general framework. Here, we simulate two cases using the SWOT raw data simulator [30]. In the first case, a specular point as a second transmit reflectarray (similar to the case described in Section 3.2) is simulated by running two separate simulations, one with the regular SWOT geometry and the second with the transmit antenna at the location of the specular source. The received voltage for the second case is scaled to −57 dB below the voltage level of the simulation with regular SWOT geometry, i.e., multiplied by a factor equal to $\log(\epsilon_{1}/a_{1})=-57/20$. The two received voltages are then simply added together. The phase screen model for this case can be written (similar to (17)) as

(30) $$\begin{array}{c} \phi_{mp}=\tan^{-1}\left\{\frac{\epsilon_{1}/a_{1}\sin\left(kd_{m}\sin\theta \right)}{1+\epsilon_{1}/a_{1}\cos\left(kd_{m}\sin\theta \right)}\right\} \end{array}$$

where $d_{m}$ is the location of the specular point on the mast measured from the transmit reflectarray. Figure 14 shows the phase screen simulated using the SWOT simulator and the phase screen model from (30). There is very good agreement between the analytical model and the simulated result. Figure 15 shows the difference between the simulation and the model which is an order of a magnitude smaller than the model itself, highlighting the good agreement between the two.

**Figure 16:**
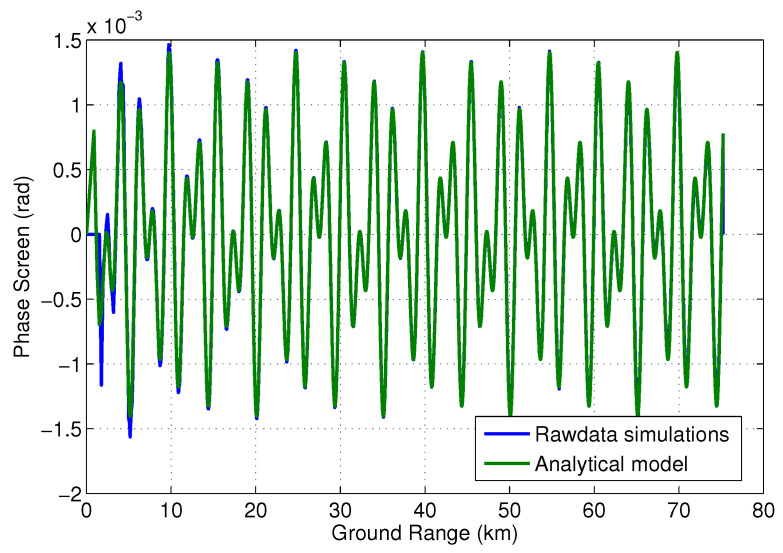
Simulated multipath for two specular points plotted along with the phase screen model given in (31).

Similarly, Figure 16 shows the comparison between model and simulated results for the second scenario with two specular sources (one at 2.5 m and one at 1 m form the baseline center). The phase screen model used here is derived from (19) and given by

(31) $$\phi_{mp}=\tan^{-1}\left\{\frac{\epsilon_{1_{1}}/a_{1}\sin\left(kd_{m_{1}}\sin\theta \right)+\epsilon_{1_{2}}/a_{1}\sin\left(kd_{m_{2}}\sin\theta \right)}{1+\epsilon_{1_{1}}/a_{1}\cos\left(kd_{m_{1}}\sin\theta \right)+\epsilon_{1_{2}}/a_{1}\cos\left(kd_{m_{2}}\sin\theta \right)}\right\}$$

where $d_{m_{i}}$ is the location of the *i*th specular point and $\epsilon_{1_{i}}/a_{1}=0.5\epsilon_{1}/a_{1}$. As we can see, there is very good agreement between the simulated result and the analytical model as well. While not exactly the case described in (19), we can use this good agreement as a validation of the framework presented thus far.

### 4.1. Variability in the Location of the Specular Points

While it may be possible to calibrate a phase screen that does not change, any change in the location of the specular points, even by a small amount, will likely alter the measured interferometric phase that cannot be calibrated, resulting in the error in height estimates to increase and affect overall instrument performance.

**Figure 17:**
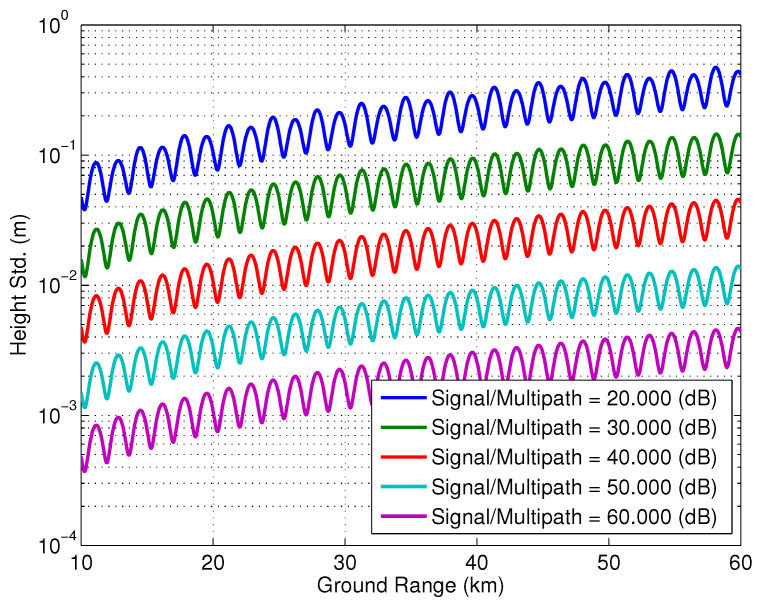
The effect of the change in the location of the specular point that acts as a second transmit reflectarray.

**Figure 18:**
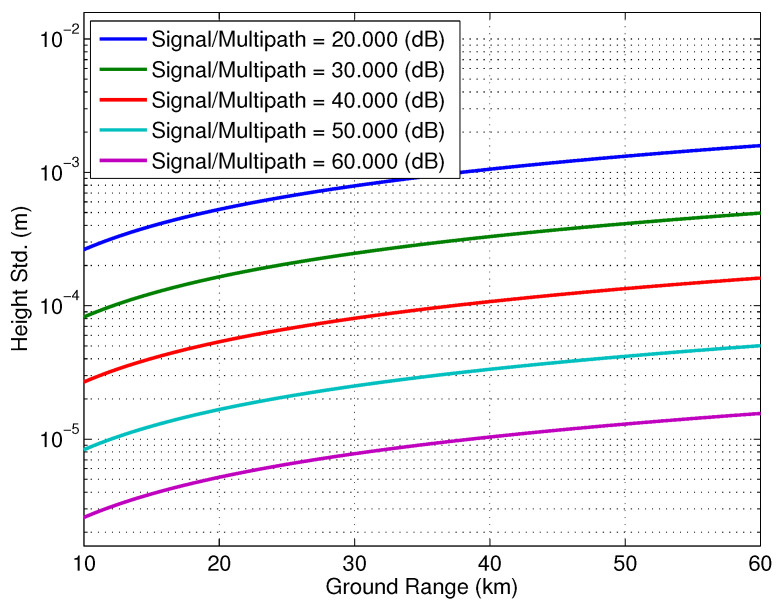
The effect of the change in the location of the specular point between the feed and reflectarray for various signal to multipath ratios.

Figure 17 shows the effect of the change in the location of the specular point that acts as a second transmit reflectarray on the multipath phase for various levels of the signal/multipath ratio. The location of this specular point is changed by 1 mm in Monte-Carlo simulations. The height std is estimated by calculating the standard deviation of the phase screen normalized by the vertical wavenumber, $k_{z}\approx kb/C$ (for a cross-track distance *C*). Here, it is assumed that $\epsilon_{2}=0$ in (17) to evaluate worst case multipath for this scenario. Figure 18, on the other hand, shows the effect of the change in location of the specular point between the feed and reflectarray. As in the previous multipath scenario, Monte-Carlo simulations were used to determine the effect of 1 mm of variation in the location of the specular point on the phase screen and, by consequence, the height estimate.

### 4.2. Electromagnetic Simulation Example

Detailed analysis of the SWOT multipath issue was conducted over a three-year period. Full electromagnetic simulations (EM) using physical optics, geometric optics and method-of-moment techniques with custom system level interferometric measurement models were used to determine the impact of various satellite outboard structures on multipath phase, as well as its sensitivity to on-orbit temperature variations and expected structural deformations.

**Figure 19:**
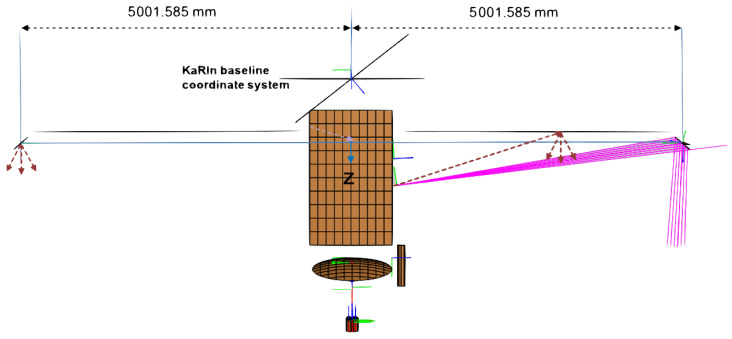
Approximate CAD model of the SWOT structure used in detailed EM simulations used to evaluate the impact of various spacecraft structures on interferometric phase.

**Figure 20:**
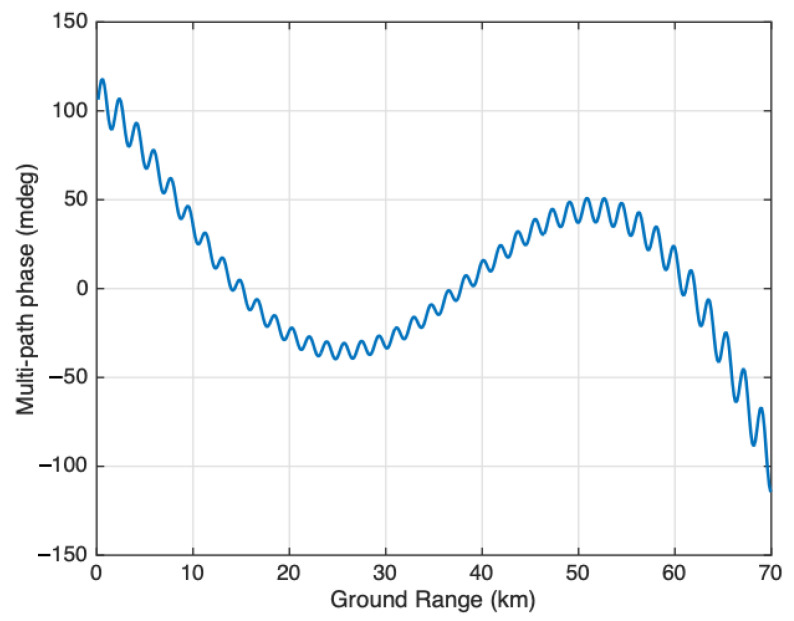
Estimated multipath phase from an initial simulation of scattering off spacecraft structure, particularly the deployed antenna mast.

Figure 19 shows, for instance, an early version of the CAD model used in the SWOT multipath study with the two 4.3 m rigid masts approximated by a perfectly conducting (PEC) plate each. While this particular version of the model does not include all the structural details of the SWOT spacecraft, it did however include all the major structural elements expected to contribute to multipath scattering. This model eventually evolved to include many more structural elements and details. Figure 20 shows the expected multipath phase for one of the two SWOT swaths computed using EM simulations and system analysis tools. The multipath phase has two distinct sinusoids, similar to the model presented in (19), describing multiple specular scattering points on the transmitter side of the spacecraft. Inversions of the simulated multipath phase based on this model suggested two scattering points, one approximately 4.2 m from the transmit antenna and one 0.1 m from the antenna. These turned out to be locations of two edges of the approximating PEC plate, an artifact of the approximate CAD model.

The structural model evolved during the multi-year study of the multipath issue, and while the details of that analysis are outside the scope of this article, the models presented here were particularly insightful in two crucial aspects. First was to help interpret the results of detailed EM simulations, pointing out various points on the structure that were acting as point scatterers, as in the example above. Modifications were made to the SWOT spacecraft to limit the contribution of those structures to the overall multipath phase. Secondly, Monte-Carlo simulations, such as those presented in Figure 17 and Figure 18, were used to determine the impact of multipath phase contributions that could vary with temperature over orbits, for instance, due to distortions in the shape of the outboard insulation (MLI), and hence be practically impossible to calibrate. This was especially important with the very strict phase stability requirements of the SWOT ocean height measurement and its coupling with orbital temperature variation. These curves, along with sensitivities determined from EM simulations, were used to derive requirements on mechanical stability of multipath mitigation structures and attenuation requirements of absorbers placed on several expected multipath hot-spots.

## 5. Conclusions

The simplified analytical models for multipath and leakage for single-pass SAR interferometers presented here are intended to provide insight into the observed interferometric phase that usually includes a combination of the undesired, albeit unavoidable error sources. These models are useful for interpreting interferometric data as well as guiding instrument design by outlining the type of signatures expected from each source. If, for instance, there is a single dominant specular reflection point on the spacecraft structure, it will show up as a sinusoid in the interferometric phase. This was indeed borne out during extensive analysis of the SWOT multipath issue, where edges of the modeled mast structure behaved as specular scatterers and the simulated multipath matched predictions of the analytical models summarized here. As the number of specular points increase, analytical models become less helpful; however, spectral analysis of the predictions was particularly insightful since the frequency of each sinusoid in the multipath phase carries information on the location of the corresponding scatterer. Analytical expressions for multipath in the spectral domain do not lead to closed form solutions; however, the spatial models presented here are sufficient for capturing the relevant impact. While multipath phase from specular points is the most likely and problematic culprit for most single-pass interferometers, for SWOT in particular, spacecraft structures redirecting energy from the feed to the reflectarray were expected to introduce multipath as well. Analytical models were also developed for this case which show a multipath phase signature independent of incidence angle and therefore more likely to be estimated on-orbit and calibrated out. Extensive EM-simulations (using physical optics, geometric optics and method of moments) were then conducted to more accurately ascertain the impact of multipath signatures from the SWOT spacecraft on interferometric phase and were used to design external structures and place absorber materials in specific locations to manage and mitigate multipath. Another important aspect of the analysis on multipath for SWOT was to determine the variability in multipath phase over varying conditions in orbit, in particular, due to thermally induced changes in MLI. This effect becomes particularly significant at Ka-band with very tight measurement accuracy requirements, as is the case with SWOT. The analytical multipath models developed here were used to determine variability in interferometric multipath phase due to changes in point scatterer locations, as a way of quantifying the effect of MLI deformation. For SWOT, even millimetric variations in MLI between the feed and antenna resulted in excessive height errors. EM-simulations that capture the full extent of random variations in MLI were impractical; however, it was fairly straightforward to use analytical multipath models in Monte-Carlo simulations to bound the error, providing critical information for key design decisions.

## Figures and Tables

**Figure A1:**
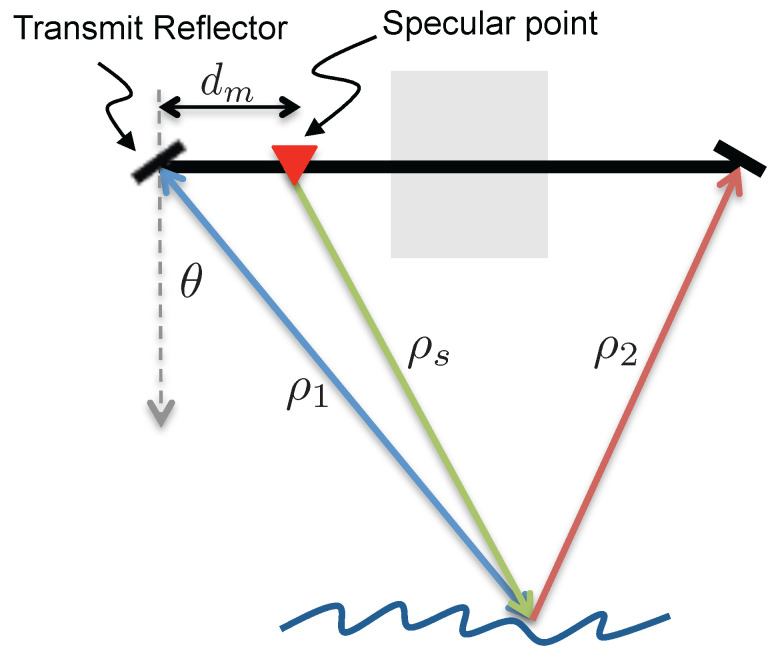
Illustration of received signal paths for a simplified singe point scatterer geometry used in the example derivation. The framework presented in this section is readily extendable to the more complex geometries discussed in the body of the article.

## Abbreviations

The following abbreviations are used in this manuscript:

SWOT	Surface Water and Ocean Topography
SAR	Synthetic Aperture Radar
GPS	Global Positioning System
DORIS	Détermination d’Orbite et Radiopositionnement Intégré par Satellite
KaRIn	Ka-Band Radar Interferometer
MLI	Multi-layer insulation
RF	Radio Frequency
EM	Electro-Magnetic
PEC	Perfect Electric Conductor
CAD	Computer Aided Design

## Appendix A. Derivation of Multipath Phase

The following is an example derivation of a simplified point-source SWOT multipath model. This general framework can be used to derive any of the models presented in this article. The basic geometry for this scenario is described in Figure A1 for a point source at a distance $d_{m}$ from the transmit reflectarray.

We begin with the simple description of the received fields at the two antennas

(A1) $$\begin{array}{cc} s_{1} & =ae^{-j2k\rho_{1}}+\epsilon_{1}e^{-jk\left(\rho_{s}+\rho_{1}\right)}\\ s_{2} & =ae^{-jk\left(\rho_{1}+\rho_{2}\right)}+\epsilon_{2}e^{-jk\left(\rho_{s}+\rho_{2}\right)} \end{array}$$

The interferometric coherence is then given by

(A2) $$\begin{array}{cc} \gamma & =s_{1}s_{2}^{*}\\ \; & =\left(ae^{-j2k\rho_{1}}+\epsilon_{1}e^{-jk\left(\rho_{s}+\rho_{1}\right)}\right){\left(ae^{-jk\left(\rho_{1}+\rho_{2}\right)}+\epsilon_{2}e^{-jk\left(\rho_{s}+\rho_{2}\right)}\right)}^{*}\\ \; & =a_{1}^{2}e^{-jk\left(2\rho_{1}-\rho_{1}-\rho_{2}\right)}+a\epsilon_{1}e^{-jk\left(\rho_{s}+\rho_{1}-\rho_{1}-\rho_{2}\right)}+a\epsilon_{2}e^{-jk\left(2\rho_{1}-\rho_{s}-\rho_{2}\right)}\\ \; & \;\;\;\;\;+\epsilon_{2}\epsilon_{1}e^{-jk\left(\rho_{s}+\rho_{1}-\rho_{s}-\rho_{2}\right)} \end{array}$$

Let us assume the contribution of the term with weighting $\epsilon_{1}\epsilon_{2}$ is negligible, then this simplifies to

(A3) $$\begin{array}{cc} \gamma & \approx a_{1}^{2}e^{-jk\left(\rho_{1}-\rho_{2}\right)}+a\epsilon_{1}e^{-jk\left(\rho_{s}-\rho_{2}\right)}+a\epsilon_{2}e^{-jk\left(2\rho_{1}-\rho_{s}-\rho_{2}\right)}\\ \; & =a_{1}^{2}e^{-jk\left(\rho_{1}-\rho_{2}\right)}\left(1+\frac{\epsilon_{1}}{a}e^{-jk\left(\rho_{s}-\rho_{2}-\rho_{1}+\rho_{2}\right)}+\frac{\epsilon_{2}}{a}e^{-jk\left(2\rho_{1}-\rho_{s}-\rho_{2}-\rho_{1}+\rho_{2}\right)}\right)\\ \; & =a_{1}^{2}e^{-jk\left(\rho_{1}-\rho_{2}\right)}\left(1+\frac{\epsilon_{1}}{a}e^{-jk\left(\rho_{s}-\rho_{1}\right)}+\frac{\epsilon_{2}}{a}e^{-jk\left(\rho_{1}-\rho_{s}\right)}\right). \end{array}$$

We define multipath phase as the difference between the actual interferometric phase and the nominal interferometric phase, such that

(A4) $$\begin{array}{c} \phi_{mp}=∠\left(\gamma \cdot \gamma {*}_{nom}\right) \end{array}$$

where the nominal coherence (without the point source), $\gamma_{nom}=a_{1}^{2}e^{-jk\left(\rho_{1}-\rho_{2}\right)}$, we obtain

(A5) $$\begin{array}{c} \phi_{mp}=∠\left(1+\frac{\epsilon_{1}}{a}e^{-jk\left(\rho_{s}-\rho_{1}\right)}+\frac{\epsilon_{2}}{a}e^{-jk\left(\rho_{1}-\rho_{s}\right)}\right). \end{array}$$

Since we can approximate $k\left(\rho_{1}-\rho_{s}\right)=kd_{m}\sin\theta$, this simplifies as

(A6) $$\begin{array}{cc} \phi_{mp} & =∠\left(1+\frac{\epsilon_{1}}{a}e^{jkd_{m}\sin\theta }+\frac{\epsilon_{2}}{a}e^{-jkd_{m}\sin\theta }\right)\\ \; & =∠\left(1+\frac{\epsilon_{1}}{a}\left(\cos\left(kd_{m}\sin\theta \right)+j\sin\left(kd_{m}\sin\theta \right)\right)\right.\\ \; & \;\;\;\;\left.+\frac{\epsilon_{2}}{a}\left(\cos\left(kd_{m}\sin\theta \right)-j\sin\left(kd_{m}\sin\theta \right)\right)\right)\\ \; & =∠\left(1+\left(\frac{\epsilon_{1}+\epsilon_{2}}{a}\right)\cos\left(kd_{m}\sin\theta \right)+j\left(\frac{\epsilon_{1}-\epsilon_{2}}{a}\right)\sin\left(kd_{m}\sin\theta \right)\right)\\ \; & =\tan^{-1}\left(\frac{\left(\epsilon_{1}-\epsilon_{2}\right)\sin\left(kd_{m}\sin\theta \right)}{a+\left(\epsilon_{1}+\epsilon_{2}\right)\cos\left(kd_{m}\sin\theta \right)}\right). \end{array}$$

## References

[1] Fu L.L., Ferrari R. (2008). Observing Oceanic Submesoscale Processes From Space. Eos Trans. Am. Geophys. Union.

[2] Fu L.L., Alsdorf D., Rodriguez E., Morrow R., Mognard N., Lambin J., Vaze P., Lafon T. The SWOT (Surface Water and Ocean Topography) Mission: Spaceborne Radar Interferometry for Oceanographic and Hydrological Applications. Proceedings of the OceanObs’09: Sustained Ocean Observations and Information for Society.

[3] Durand M., Fu L.L., Lettenmaier D.P., Alsdorf D.E., Rodriguez E., Esteban-Fernandez D. (2010). The Surface Water and Ocean Topography Mission: Observing Terrestrial Surface Water and Oceanic Submesoscale Eddies. Proc. IEEE.

[4] Vaze P.V., Neeck S.P., Martimort P., Kimura T. (2019). SWOT: Development of the wide-swath surface water altimetry mission for oceanography and hydrology (Conference Presentation). Proceedings of the Sensors, Systems, and Next-Generation Satellites XXIII.

[5] Graham L.C. (1974). Synthetic interferometer radar for topographic mapping. Proc. IEEE.

[6] Zebker H.A., Goldstein R.M. (1986). Topographic mapping from interferometer synthetic aperture radar observations. J. Geophys. Res..

[7] Rodriguez E., Martin J.M. (1992). Theory and Design of Interferometric Synthetic Aperture Radars. Proc. Inst. Elect. Eng..

[8] Rosen P.A., Hensley S., Joughin I.R., Li F.K., Madsen S.N., Rodriguez E., Goldstein R.M. (2000). Synthetic aperture radar interferometry. Proc. Inst. Elect. Eng..

[9] Wang J., Fu L.L., Torres H.S., Chen S., Qiu B., Menemenlis D. (2019). On the Spatial Scales to be Resolved by the Surface Water and Ocean Topography Ka-Band Radar Interferometer. J. Atmos. Ocean. Technol..

[10] Fu L.L., Morrow R. Observing the Ocean Surface Topography at High-Resolution by the SWOT (Surface Water and Ocean Topography) Mission. Proceedings of the IGARSS 2018—2018 IEEE International Geoscience and Remote Sensing Symposium.

[11] Ma C., Guo X., Zhang H., Di J., Chen G. (2020). An Investigation of the Influences of SWOT Sampling and Errors on Ocean Eddy Observation. Remote Sens..

[12] Morrow R., Fu L.L., Ardhuin F., Benkiran M., Chapron B., Cosme E., d’Ovidio F., Farrar J.T., Gille S.T., Lapeyre G. (2019). Global Observations of Fine-Scale Ocean Surface Topography With the Surface Water and Ocean Topography (SWOT) Mission. Front. Mar. Sci..

[13] Gaultier L., Ubelmann C., Fu L.L. (2016). The Challenge of Using Future SWOT Data for Oceanic Field Reconstruction. J. Atmos. Ocean. Technol..

[14] Rodriguez E., Esteban-Fernandez D., Meynart R., Neeck S.P., Shimoda H. (2010). The Surface Water and Ocean Topography Mission (SWOT): The Ka-band Radar Interferometer (KaRIn) for water level measurements at all scales. Preceedings of the Sensors, Systems, and Next-Generation Satellites XIV, Toulouse, France, 20–23 September 2010.

[15] Fjørtoft R., Gaudin J.M., Pourthié N., Lalaurie J.C., Mallet A., Nouvel J.F., Martinot-Lagarde J., Oriot H., Borderies P., Ruiz C. (2014). KaRIn on SWOT: Characteristics of Near-Nadir Ka-Band Interferometric SAR Imagery. IEEE Trans. Geosci. Remote Sens..

[16] Peral E., Esteban-Fernandez D. Swot Mission Performance and Error Budget. Proceedings of the IGARSS 2018—2018 IEEE International Geoscience and Remote Sensing Symposium.

[17] Vaze P., Kaki S., Limonadi D., Esteban-Fernandez D., Zohar G. The surface water and ocean topography mission. Proceedings of the 2018 IEEE Aerospace Conference.

[18] Couhert A., Mercier F., Moyard J., Jalabert E., Houry S., Ait Lakbir H., Masson C. Contribution of DORIS in Unveiling Systematic Errors in Altimeter Satellites’ Precise Orbits. Proceedings of the 42nd COSPAR Scientific Assembly.

[19] Chae C.S. Advanced Microwave Radiometer (AMR) for SWOT mission. Proceedings of the AGU Fall Meeting.

[20] Hodges R., Zawadzki M. Ka-band reflectarray for interferometric SAR altimeter. Proceedings of the 2012 IEEE International Symposium on Antennas and Propagation.

[21] Hodges R.E., Chen J.C., Radway M.R., Amaro L.R., Khayatian B., Munger J. (2020). An Extremely Large Ka-Band Reflectarray Antenna for Interferometric Synthetic Aperture Radar: Enabling Next-Generation Satellite Remote Sensing. IEEE Antennas Propag. Mag..

[22] Fang H., Sunada E., Chaubell M.J., Esteban-Fernandez D., Thomson M., Nicaise F. Thermal deformation and RF performance analyses for the SWOT large deployable Ka-band reflectarray. Proceedings of the 51st AIAA/ASME/ASCE/AHS/ASC Structures Structural Dynamics, and Materials Conference.

[23] Farr T.G., Rosen P.A., Caro E., Crippen R., Duren R., Hensley S., Kobrick M., Paller M., Rodriguez E., Roth L. (2007). The shuttle radar topography mission. Rev. Geophys..

[24] Chapin E., Hensley S., Michel T. Calibration of an across track interferometric P-band SAR. Proceedings of the IEEE 2001 International Geoscience and Remote Sensing Symposium (Cat. No.01CH37217).

[25] Hensley S., Rosen P.A., Gurrola E. (2000). Topographic map generation from the Shuttle Radar Topography Mission C-band SCANSAR interferometry. Proceedings of the Microwave Remote Sensing of the Atmosphere and Environment II.

[26] Hensley S., Rosen P., Gurrola E. The SRTM topographic mapping processor. Proceedings of the IGARSS 2000. IEEE 2000 International Geoscience and Remote Sensing Symposium. Taking the Pulse of the Planet: The Role of Remote Sensing in Managing the Environment. Proceedings (Cat. No. 00CH37120).

[27] Mao Y., Xiang M., Wei L., Han S. The mathematic model of multipath error in airborne interferometric SAR system. Proceedings of the 2010 IEEE International Geoscience and Remote Sensing Symposium.

[28] Pinheiro M., Prats P., Scheiber R., Fischer J. Multi-path correction model for multi-channel airborne SAR. Proceedings of the 2009 IEEE International Geoscience and Remote Sensing Symposium.

[29] JPL/NASA (2020). SWOT 3D Model. https://swot.jpl.nasa.gov/resources/86/swot-3d-model/.

[30] Peral E., Rodríguez E., Esteban-Fernández D. (2015). Impact of surface waves on SWOT’s projected ocean accuracy. Remote Sens..

